# Knowledge, Attitude, and Practice of Community towards an Onchocerciasis Elimination Program from South West Ethiopia

**DOI:** 10.1155/2022/1417804

**Published:** 2022-06-24

**Authors:** Haile Worku, Misganaw Mola, Bizuwork Derebew Alemu, Sebwedin Surur Jemal, Aklilu Ayiza, Samuel Getachew, Nitin Mahendra Chauhan, Sunil Tulshiram Hajare, Suresh Chandra Singh, Mohammed Kuddus, Vijay J. Upadhye

**Affiliations:** ^1^Department of Biology, College of Natural and Computational Sciences, Mizan Tepi University, Mizan Tepi, Ethiopia; ^2^Department of Statistics, College of Natural and Computational Sciences, Mizan Tepi University, Mizan Tepi, Ethiopia; ^3^Department of Biology, College of Natural and Computational Sciences, Dilla University, Dilla 419, Ethiopia; ^4^TB and COVID Antigen Testing Lab, Vadodara, Gujarat, India; ^5^Department of Biochemistry, College of Medicine, University of Hail, Hail, Saudi Arabia; ^6^Parul Institute of Applied Sciences, Parul University, Vadodara 391760, Gujarat, India

## Abstract

Onchocerciasis is a neglected tropical disease that is prevalent throughout Africa, including developing countries such as Ethiopia. It affects around 37 million people, the majority of whom are from Africa. As a result, the study was designed to look into the community's knowledge, attitude, and practice about the onchocerciasis elimination campaign. Four communities from Gesha town, Southwest Ethiopia, were chosen. The population was selected using a basic random selection procedure, and 312 people were identified for the study based on the eligibility requirements, with 302 (96.79%) of them responding correctly. The data were analyzed using the descriptive method with the SPSS program version 20. It was discovered that the majority of communities (89.4%) are aware of onchocerciasis. They also have a good awareness of the severity, preventability, therapy, and mode of transmission, yet they have certain misunderstandings. The communities attitude towards community directed therapy (CDT) using Ivermectin is positive (68.5%). According to 56% of the community, offering incentives for community drug distributors (CDD) has the potential to make the elimination campaign more successful. Different measures, such as avoiding any activities near the river, are important in the process of eradicating this disease at the community level. As a result, the respondent demonstrates that covering the lower body part lessens the black fly's vulnerability because they may attack below the knee. In general, community awareness and attitude are required to eradicate this disease from the district. In addition, increased stakeholder participation and offering motivating rewards for CDT are required to make the elimination program a success.

## 1. Introduction

Onchocerciasis is endemic to 34 African countries and is the second greatest cause of blindness in humans, causing severe skin and eye diseases [1]. *Onchocerca volvulus*, a filarial worm with humans as the only definitive host, causes it [2]. More than 120 million people worldwide are in danger of developing the disease, with 18 million already infected, and Africa accounts for more than 99% of the disease burden [3]. Onchocerciasis affects the working-age population and is the world's second-leading infectious cause of blindness, causing 1.15 million people to go blind [4].

In many African countries, the disease poses a public health and socioeconomic danger [5], and it is a big problem for rural communities living near rivers in Sub-Saharan Africa, Latin America, and Central America. The disease is carried by black flies of the species Simulium, which breed in fast-flowing rivers and bite people [6].

Onchocerciasis is a disease that affects poor rural populations in Ethiopia, and it is one of the underlying reasons of poverty in the regions where it is found. In 1939, Italian investigators in Southwest Ethiopia discovered onchocerciasis for the first time [7]. Since then, thorough epidemiological investigations undertaken by many professionals have established the disease's existence across the Southwestern, Western, and Northwestern areas of the country, with varying degrees of endemicity [8].

Between 1974 and 2002, onchocerciasis disease was controlled in West Africa thanks to the activities of the Onchocerciasis Control Program (OCP), which primarily involved helicopters and airplanes for spraying of pesticides against black fly larvae (vector control). Since 1989, large-scale dissemination of Ivermectin has augmented this [4].

According to a previous report from Ethiopia, onchocerciasis causes poor school performance and a high dropout rate among afflicted children, as well as severe social stigmatization, particularly among women [9]. The disease's incidence is linked to activities that expose people to vectors, such as farming, washing clothes near a river, and swimming [10]. Aside from the clinical consequences, the disease has a social and economic impact on individuals and entire communities [11]. Gesha zone is one of the towns in Kafa zone that is known to be endemic for onchocerciasis [12]. Kafa zone is one of the zones located in Southern Nation Nationalities and People's Regional state that is known to be endemic for onchocerciasis.

The current state of a disease and its effects on society's well-being, as well as the possibility that it is related to onchocerciasis, inadequate knowledge among affected individuals, and ineffective key stack holders, are disturbing causes for the researcher. Many years of Ivermectin treatment are required to achieve elimination in an onchocerciasis endemic area [13]. Despite the fact that there was widespread medicine administration in this area for several years, the disease could not be eradicated [14]. Onchocerciasis elimination in Africa would have significant health and economic benefits, reducing the demand for health workers and outpatient services. To achieve these benefits, community, national, and global policymakers would need to work together and promote eradication [15].

The absence of a major study in the specified topic at the selected area, in addition to the aforesaid starting points, is the basis for doing the predicted research. As a result, the goal of this study is to discover the significant gap between stack holders knowledge, attitude, and practice about the elimination of onchocerciasis in the community. It will aid in the elimination of onchocerciasis in practice and be used to determine whether present intervention tactics are effective or whether further intervention strategies are required to reach the Ethiopian Ministry of Health's aim. In addition, raise community awareness about the consequences of onchocerciasis. Additionally, this study may serve as a resource for anyone interested in conducting further research, particularly in the area of Neglected Tropical Diseases (NTD). In the last, the CDD knowledge of onchocerciasis and attitudes regarding the CDT has not been investigated in the studied area. Therefore, the goal of this study was to look into CDD knowledge and views regarding onchocerciasis, as well as their attitudes about the CDT, in the Gesha town of South-Western Ethiopia.

## 2. Materials and Methods

### 2.1. Description of the Study Area

Gesha town is found in the Kaffa zone, which is at a distance of 571 kilometers far away from Addis Ababa in South West Ethiopia. The town has a total number of 25 villages. Based on the 2007 census report of CSA, the town had a total population of 85,104, of whom 41,441 were men and 43,663 were women. Of the total population, 3,433 (4.03%) are urban dwellers [16]. CDT program was introduced to Gesha town in 2001 by WHO/APOC. There are 6 rivers, namely Gonogory, Datay, Yobateshe, Shewleche, Ginnay, and Ocashy, were found in the studied town. The town consists of 4 health centers that provide routine services for the population. Onchocerciasis is one of the major public health problems in the town.

### 2.2. Study Design

A community-based cross-sectional study was conducted from January 2020 to June 2020 in Gesha town to determine knowledge, attitudes, and practice of the community toward an onchocerciasis elimination program. Both quantitative and qualitative data were collected from each selected village.

### 2.3. Sampling Technique and Sample Size

Out of the 25 villages, two villages were randomly selected, namely, Meshami and Abeta, while the remaining two villages, mainly Deka and Kicho from the Gesha town, were purposely selected based on their location nearer to the Shewuleche River. During data collection, Ivermectin treatment registration book of each village was used as the sampling frame. Based on the Ivermectin treatment registration book, the household of each village were as follows: Meshami (350), Deka (615), Abeta (200), and Kicho (250). Therefore, the total population size of the study area was 1415. Since the total population size is known, the final sample size was calculated without overestimating at 95% confidence and 5% degree of accuracy. Thus, the sample size for collecting quantitative data from four selected village was determined using a simplified formula of Yamane [17] for known population size as follows:

(1) $$\begin{array}{c} n=\frac{N}{1+N{\left(e\right)}^{2}}, \end{array}$$

where *n* is the sample size; *N* is the population size, and *e* is the level of precision [18]. From each village, the respondents were selected by using the following formula:

(2) $$\begin{array}{c} n=\frac{\text{NHT}\times \text{TSHH}}{N}, \end{array}$$

where *n* is the number of households to be involved in the questionnaire; NHT belongs to the number of households in a subdistrict; *N* is the total number of households in the district, and TSHH represents the total number of sample households from the district. The sample size for each selected place was calculated as follows:

(3) $$\begin{array}{c} n=\frac{350\times 312}{1415}=77. \end{array}$$

Based on the above formula, the total number of respondents from Meshami, Deka, Abeta, and Kicho includes 77, 135, 45, and 55, respectively, were involved in a questionnaire based interviewing. To select the individual respondents from each village, a stratified sampling technique was applied. The communities are divided into groups according to a common attribute and a random sample was selected within each group. To get reliable information for health workers from each two health centres of Meshami and Deka, one health extension worker and two drug distributors were selected from each selected village by using the purposive sampling technique because they were assumed to be rich in the information required. The respondents who were selected using purposive sampling are a member of the preestimated sample size. In this case, some grouping of the population was considered (age, sex, and area). The participants were eligible if they were a member of the selected village, age 18 years and above, apparently healthy, and willing to volunteer to participate in the study. Based on the number of people aged 18 years and above in each village, the preestimated sample size of 312 was proportionally distributed. Study participants (those whose age was above 18 years and height greater than or equal to 90 cm) were recruited using simple random sampling based on Ivermectin registration book.

### 2.4. Data Collection Instrument and Procedure

Structured questionnaires were prepared in English based on information from available literature and the questionnaires were translated into the Amharic language. A total of 302 questionnaires were distributed and 100% completed questionnaires were returned from four villages. The questionnaire contains four parts that include five items. The first part includes demographic questions, the second part includes knowledge questions, the third part consists of attitude, and the fourth part constitutes community practices to eliminate onchocerciasis. The participants were surveyed in their local language by trained data collectors (health extension) who speak the local language. Each questionnaire was made by a house-to-house visit using a stratified sampling technique.

In this study, Cronbach's alpha coefficients were used to assess the reliability of the instrument. Cronbach's alpha coefficient is a measure of internal consistency. The quantitative data were interpreted in the form of graphs and tables, while the qualitative data was interpreted in descriptive form by using descriptive statistical tools such as percentage and mean consistency based on all the possible correlations between the items within the scale. The reliability of the instruments was Cronbach's alpha value of 0.66 for questionnaires, which indicated the high internal consistency [19].

### 2.5. Data Analysis

The data was transferred to SPSS software version 20 and analyzed according to the different variables. The Pearson Chi-square was used to evaluate the statistical significance of the bivariate association of the selected covariant. Both logistic regression analysis and binary logistic regression were referred to in order to determine the association between a categorical dependent variable and a set of independent (explanatory) variables using crude odds ratio with a corresponding 95% confidence interval (CI). To control possible confounders, variables that exhibited statistical significance during bivariate analysis with a *P* value ≤0.25 were incorporated into multivariate logistic regression analysis.

### 2.6. Ethical Considerations

Permission was received from Mizan Tepi University, the Gesha town health office, and the four administrators of the designated villages. The study's aims were explained to the participants, and they were guaranteed that the data would be kept private. Prior to data collection, all subjects gave verbal informed consent.

## 3. Results

### 3.1. Characteristics of Study Respondents

Among the 312 study participants, 178 (58.9) were male, and 87 (28.8) were in the age group of 18–25 years. In this study, most of the respondents 230 (76.1%)) were found in the age group of 18 to 45 years. Out of the study respondents, 157 (52%) of them were illiterate, and 49 (16.2%) of them attended secondary school education. 144 (47.7%) and 78 (25.8%) study cases were farmers and students, respectively (Table 1). Out of 312 respondents, 302 (96.79%) of the respondents provided their response properly, whereas 3.21% of the respondent unable to provide their response properly. As shown in Table 1, with regard to occupation out of 312 respondents, 144 (47.7%) of them were farmers, 22 (7.3%) of them were teachers, 22 (7.3%) of them were health workers, 31 (10.3%) of them were merchants, 78 (25.8%) of them were students, and 5 (1.7) of them belonged to different occupations.

### 3.2. Knowledge of Respondents about Onchocerciasis

Nearly half of the respondents, 179 (59.3%), have agreed that onchocerciasis is transmitted from person to person, while 62 (20.5%) respondents have disagreed with this idea. The rest include 61 (20.2%) of respondents who are not sure about the transmission of the disease from person to person. Almost all of the respondents have agreed that this disease needs treatment 278 (92.0%) and also the majority of the respondents have agreed that this disease is preventable 262 (86.8%). About 277 (91.72%) of the study participant agreed that onchocerciasis is a serious disease, and 14 (4.56) disagreed with this idea. 3.64% of the respondents were neutral. As shown in Table 2, about 270 (89.4%) study participants have heard about onchocerciasis and locally named it as “Foket.” The remaining 32 (10.6%) had no information about onchocerciasis. Most of them, 213 (70.5%), had not ever been sick with the disease. However, 89 (29.5%) have been sick with it previously.

### 3.3. Knowledge of Respondents on the Cause and Treatment of Onchocerciasis

As shown in Table 3, respondents have a good understanding of the mode of transmission way of onchocerciasis. As the study illustrated, 223 (73.8%) of the respondent know that onchocerciasis is transmitted from person to person by the black fly bite. On the other hand, 32 (10.6%) of the sampled community member held various misconceptions about the way of onchocerciasis transmission. Out of 302 respondents, 47 (15.6%) have no knowledge of whether the disease is communicable or not. In spite of these, the study participants can not differentiate the vector and the parasite of this disease. Few of the respondent, such as 71 (23.5%), has knowledge of the causative agent of onchocerciasis, which is *Onchocerca volvulus*. But 75% of the study participants considered a black fly as both the disease transmitter (vector) and the etiology (parasite) of the disease. Almost the entire respondents mentioned that this disease is treated by modern medicine. Of 302 respondents, 231 (76.5%) participants admitted to having used the drug Ivermectin to eliminate this disease. Whereas 52 (17.2%) of participants did not know the exact name of the drug instead, they knew the drug name as “oncho” in their locality. As determined in Table 3, 230 (76.4%) of respondents has informed about the elimination program of onchocerciasis. Out of the respondents, 71 (23.6%) do not have information about the elimination strategies. 192 (79.3%) agreed that the information about the elimination program of this disease can mainly be obtained from health extension workers because they have close relations with society. About 38 (15.7) and 11 (4.5) of the respondents get the information from CDD and mass media, respectively.

### 3.4. Attitude of Community Respondents about Onchocerciasis

From Table 4, among 302 respondents, 207 (68.5%) perceive the community CDT with the Ivermectin program is very useful, but 31.1% of respondents perceive the program as partially useful, and 3% perceive that the program is not useful. Nearly half of the respondents (165 (54.6%)) believed that the drug that treats this disease has side effects. Others, 137 (45.4%) respondents, understood that the drug has no side effects. 242 (80.1%) of the respondent believed that the controlling program of onchocerciasis is effective in their locality. However, 38 (12.6%) of the respondents pointed that the elimination program is not effective and it needs extra effort to eradicate ultimately. To make the program very effective, 56.6% of the respondents recommended that providing incentives for CDD is mandatory. In accordance with this, 10.3% of respondents illustrated that creating community awareness also has a great role in the success of the elimination of this disease.

### 3.5. Practice of the Community towards the Success of Elimination of Onchocerciasis

Table 5 revealed the summary of the practice of the community to eliminate onchocerciasis in their locality. During drug distribution, 169 (56%) of the respondents provided information that there was a problem with following onchocerciasis treatment properly. This is due to mainly 198 (65.6%) having no incentive for CDD. As the respondent revealed that the drug is delivered to the local community on the basis of height. However, due to lack of incentive for CDD, the drug is now distributed without considering this measure and sometimes they allow for drug users to take the drug to their home and this leads to missuse of the drug. As the health extension worker pointed out, orally infrequently, few peasants were observed when they applied this drug to the fattening of cattle informally. Consequently, the respondents have shown that considering this disease as a sign of the anger of God (8.6%), believing freely given medicine as useless (14.2%) and fear of side effects of the drug (23.5%) creates challenges during treatment. While the remaining 131 (43.4%) respondents provided that there was no problem during drug distribution. Data on Table 5 realized that majority 181 (67.8%) of the study participant has taken the last treatment in this year (2019). This indicates the disease is still also prevalent in the district. Some members of the community missed the treatment. Respondents have missed the treatment in relation to pregnancy (33.1%), health problems (21.2%), and being absent during the drug distribution period (45.7%). In addition to this, some of the respondents mentioned in the interview part are people who usually ignore the drug deliberately without any tangible reason. This may be a lack of awareness about the drug.

**Figure 1:**
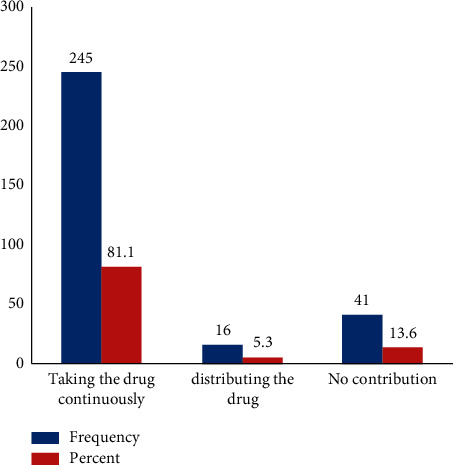
Contribution of the community towards the success of CDT with Ivermectin from Gesha town, Ethiopia.

According to the respondent response majority, 80.1% of the community contributes to the CDT by taking Ivermectin drugs. 13.6% of the respondents said that they have no contribution to the CDT program with respect to Ivermectin. From all the participants studied, 5.3% played a role in distributing the drug (Figure 1).

### 3.6. Prevention Measures Taken by Study Community for Elimination of Onchocerciasis

**Figure 2:**
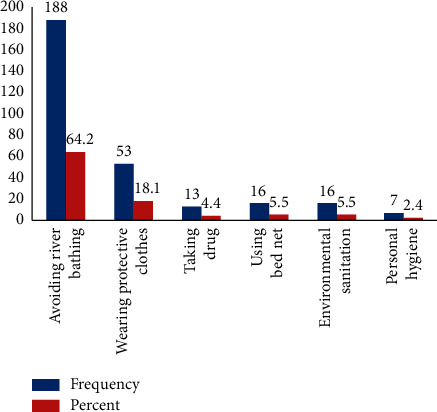
Prevention measure taken by the community towards the elimination of onchocerciasis from Gesha town, Ethiopia.

Even if the community prevents themselves from being infected with this disease by avoiding river bathing 188 (64.2%), other participants have a misconception about the way of prevention measures. According to Figure 2, people believe 18.1% wear a protective cloth, 4.4% take drugs, 5.5% use net, 5.5% use environmental sanitation, and 2.4% personal hygiene is assumed to prevent them from biting of black flies. Some respondents, 110 (63.6%), illustrated that covering the lower extremities (below the knees) during the work around the river is important to block the bite of the fly. Other respondents mentioned in the interview part of this study that the black fly mainly bites below the knee. Some members of the community have a misconception about how to prevent themselves from being infected. For instance, some believe that environmental sanitation and personal hygiene may prevent equally 5.3% of this disease.

### 3.7. Bivariate Binary Logistic Regression Analysis of Knowledge, Attitude, and Practice towards Elimination of Onchocerciasis

Educational status, age of respondent, sexes of respondent, and employment of respondent all revealed statistical differences in knowledge of the onchocerciasis elimination program (*P* < 0.25). Male respondents had a lower chance of knowing about the onchocerciasis elimination program than female respondents. When compared to respondents aged 18–25, participants aged 26–35, 36–45, and 46–55 were less likely to know about the onchocerciasis elimination program. Educational status, age of respondent, sex of respondents, and employment of participants were statistically linked with attitude toward the onchocerciasis elimination program (*P* < 0.25). Lastly, educational status, respondent age as well as sex, and participant's occupation were statistically linked with practice toward the onchocerciasis elimination program (*P* < 0.25) (Table 6).

### 3.8. Multivariable Binary Logistic Regression of Knowledgeable Factors Associated with Elimination of Onchocerciasis

According to a multivariable binary logistic analysis, being female, being between the ages of 26 and 35, being between the ages of 46 and 55, having a primary school education, having a diploma, and being a civil servant were all found to be significant factors in the knowledge of the onchocerciasis elimination program in Gesha, Ethiopia (Table 7). Male respondents were 0.04 times (95% CI: 0.09–0.178) less likely than female respondents to know about the onchocerciasis elimination program. When compared to respondents in the 18–25 age group, those in the 26–35 age group were 0.016 times (95% CI: 0.01–0.057) less likely to be aware of the onchocerciasis elimination program. When compared to respondents in the 18–55 age group, those in the 46–55 age group were 0.012 times (95% CI: 0.00–0.365) less likely to know about the onchocerciasis elimination program.

### 3.9. Multivariable Binary Logistic Regression of Attitude towards Elimination of Onchocerciasis

According to a multivariable binary logistic analysis, being a man between the ages of 26 and 35, attending primary school, and being illiterate were all significant predictors in attitude toward an onchocerciasis elimination program in Gesha, Ethiopia (Table 8). Males were 0.258 times (95% CI: 0.119–0.561) less likely than females to support an onchocerciasis elimination program. When compared to respondents in the 18–25 age group, individuals in the 26–35 age group were 0.276 times (95% CI: 0.84–0.902) less likely to have a favorable attitude toward the onchocerciasis elimination program. Furthermore, illiterate respondents had a 0.007 (95% CI: 0.001–0.057) times lower odds of having a good attitude toward an onchocerciasis elimination program than those with a Bachelor's degree or higher. The research also discovered that respondents who attend degree and above hold an effect over other covariates fixed (Table 8).

### 3.10. Multivariable Binary Logistic Regression of Practicable Factors towards Elimination of Onchocerciasis

Male gender, illiteracy, and attendance at elementary schools were found to be significant determinants in onchocerciasis elimination practices in Gesha, Ethiopia, according to multivariable binary logistic analysis (Table 9). Males were 0.42 times (95% CI: 0.180–0.900) less likely than females to participate in an onchocerciasis elimination program. Furthermore, uneducated respondents had a 0.114 (95% CI: 0.45–0.292) times lower odds of having good practice toward an onchocerciasis elimination program than those with a Bachelor's degree or higher. The study also found that respondents with a diploma were 0.166 (95% CI: 0.049–0.442) times less likely to be aware of an onchocerciasis elimination program than those with a Bachelor's degree or higher when all factors were held constant (Table 9).

## 4. Discussion

The proposed study examined community respondents' knowledge, attitudes, and practices regarding the elimination of onchocerciasis. The bulk of the respondents, or 89.4%, had heard of the disease known as onchocerciasis. This research aligns with Yirga et al.'s [20] study in Bebeka, Southwest Ethiopia, where they stated verbally that they do not know the right term for the condition. The result was also backed up by many studies conducted in Ethiopia's various regions. According to Dana et al. [21] and Yirga et al. [20], nearly half of the respondents, 147 (48.7%), strongly agree that onchocerciasis is transferred from person to person, while 32 (10.6%) disagree. The remaining responders, 123 (40.7%), oppose disease transfer from person to person.

Our findings were also in agreement with those of Weldegebreal et al. [22] and Dunn et al. [23]. More than 75% of the study participants were classified as black flies, which are both a disease transmitter (vector) and a disease cause (parasite). According to Yirga et al. [20], the public has a misunderstanding about the cause of this disease. Almost every response said that modern medication is used to cure this ailment. Out of 302 individuals, 231 (76.5%) admitted to using the medicine Ivermectin to treat this condition, which was shown to be identical to Kim et al. findings [15]. The Pearson correlation between the distance of the river from the house and the degree of infection was −0.612, according to the results. As a result, the negative sign indicated that as the community settles closer to the river, the level of infection rises, and vice versa. This is in agreement with the findings of Enk et al. [24], who said that the level of infection is highest in areas near rivers.

The medicine Ivermectin has been discovered to have serious side effects, including skin allergies. The findings of Tielsch and Beech [25] corroborate the conclusion. According to the results of this survey, 76.5% of respondents reported using the medicine Ivermectin twice a year to treat this condition. This result is consistent with Kim et al. findings [15]. However, according to another study, OEPA (Onchocerciasis Elimination Program of the Americas) is now using doxycycline and Ivermectin treatment four times a year as a strategy to ensure the final removal of the parasite [26]. The major ways to maintain the eradication program, according to the respondents in this study, are medicine supply (56.6%) and providing incentives for CDD (25.5%). The findings of Yirga et al. [20] and Surakat et al. [27], respectively, support this hypothesis. During drug distribution, 169 (56%) of respondents stated that they were having difficulty following onchocerciasis treatment adequately. This is owing to the fact that 198 (65.6%) of community drug distributors have no incentive (CDD). Surakat et al. [27] conducted a study in Nigeria, while Woldegebriel et al. [6] conducted a study in Quadra, North West Ethiopia. Furthermore, respondents revealed that perceiving this ailment to be a sign of God's wrath (8.6%), believing freely offered medicine to be meaningless (14.2%), and dread of drug side effects (23.5%) all pose problems throughout treatment. It reminds me of Makenga et al.'s work [28].

Participants in the study discovered that 46.9% of community members missed out on the drug because they were not there during drug distribution and were forced to take Ivermectin. Meribo et al. [11] conducted a study similar to this. In addition, certain people of the community have been missing out on the treatment. For example, some of them have missed work due to pregnancy, health issues, or being unavailable during the medication delivery period. It is backed up by Emukah et al. findings [14]. As the respondents pointed out, raising community awareness about the disease's cause and transmission route may help to lessen the number of people who are affected by it. This is identical to Meribo et al. [11] finding result. The majority of respondents (73.8%) believed that black flies were the disease's causal agent, which is consistent with the findings of research done in Bebeka, Southwest Ethiopia [20]. Another strategy to avoid being bitten by a black fly is to wear long clothing that extends beyond the knee because the vector prefers to infest the lower body, according to the respondent. The WHO [13] report backs up this conclusion. Although many people avoid being bitten by flies by wearing clothing that covers their legs adequately, they often complain about being uncomfortable in such clothes, especially when working on the farm, which leads them to wear shorts and get bitten by flies, exposing them to disease. This could be one of the factors contributing to the expansion of onchocerciasis in the chosen area. As a result, proper training and awareness are required at the community level in order to prevent sickness and safeguard the population.

The study's weaknesses were that it was not backed up by epidemiological studies. Despite this limitation, this study provides important information about community knowledge, attitude, and practice regarding onchocerciasis, as well as identifying community perceptions that impede the uptake of preventive and treatment services in the district and providing baseline information for the Gesha Town Health Office in planning, monitoring, and evaluation of ongoing onchocerciasis control.

## 5. Conclusion

The majority of the communities are familiar with onchocerciasis. Some members of the community have misconceptions about the disease's cause, spread, and prevention. For example, the communities are unable to distinguish between the vector and the causal agent. The majority of the community has a favorable opinion of CDT with Ivermectin. However, the success of the program was hampered by the interruption of some community members during the drug campaign. The community was both knowingly and unknowingly affected by this disruption. Without any reasonable evidence, some members of the community refuse to take Ivermectin. Moreover, some members of the community interrupted the program by believing this drug may create skin allergies. Infrequently, as the health extension worker pointed out orally, few peasants were observed when they applied this drug for fattening of cattle informally. The contribution of stakeholders is also low for the success of elimination of this disease. There was no continuous followup and product report in relation to the proper implementation of the drug. To promote the elimination program, the health workers should play a role in creating community awareness about the cause, transmission, and prevention of this serious disease. CDD must give government remuneration to improve the success of drug distribution and will help with the eradication campaign. Modern medicine is being employed as part of the community's huge attempt to eradicate this disease (Ivermectin). In order to minimize contact with the vector, the communities also avoid going swimming in the river. Finally, if CDT with Ivermectin is used four times a year and Doxycycline is used in addition to Ivermectin, like in American elimination efforts, the disease will eventually be eradicated from the district. Last but not least, the stakeholder should educate the community about the disease's impact and significance.

## Figures and Tables

**Table 1:** Major demographic characteristics of the respondents from Gesha town, Ethiopia.

Characteristics	Category	Frequency (%)
Sex	Male	178 (58.0)
	Female	124 (41.0)
Age groups	18–25	87 (28.8)
	26–35	72 (23.8)
	36–45	71 (23.5)
	46–55	39 (12.9)
	56–65	33 (10.9)
Educational status	Illiterate	157 (52.0)
	Primary school	41 (13.6)
	Secondary school	49 (16.2)
	Diploma	45 (14.9)
	Degree and above	10 (3.3)
Occupation	Farmer	144 (47.7)
	Civil servant	44 (14.6)
	Merchant	31 (10.3)
	Student	78 (25.8)
	Other	5 (1.7)

**Table 2:** Knowledge of respondents about the transmission, prevention, and treatment of onchocerciasis from Gesha town, Ethiopia.

Indicative questions	Response category	Frequency (%)
Onchocerciasis transmit from person to person	Strongly disagree	39 (12.9)
	Disagree	23 (7.6)
	Undecided	61 (20.2)
	Agree	32 (10.6)
	Strongly agree	147 (48.7)
Onchocerciasis need treatment	Strongly disagree	10 (3.3)
	Disagree	8 (2.6)
	Undecided	6 (2.0)
	Agree	40 (13.2)
	Strongly agree	238 (78.8)
Onchocerciasis is preventable	Strongly disagree	17 (5.6)
	Disagree	11 (3.6)
	Undecided	12 (3.9)
	Agree	29 (9.6)
	Strongly agree	233 (77.1)
Onchocerciasis is a serious disease	Strongly disagree	9 (2.90)
	Disagree	5 (1.66)
	Undecided	11 (3.64)
	Agree	39 (12.91)
	Strongly agree	238 (78.8)
Have you heard about onchocerciasis?	Yes	270 (89.4)
	No	32 (10.6)
Have you/your family ever been sick with onchocerciasis?	Yes	89 (29.4)
	No	213 (70.6)

**Table 3:** Knowledge of respondents on the cause and treatment of onchocerciasis from Gesha town, Ethiopia.

Items	Categories of response	Frequency (%)
How does onchocerciasis transmit from person to person?	Black flies bite	223 (73.8)
	Mosquitoes bite	15 (5)
	Through contact	8 (2.6)
	Sharing cloth	7 (2.3)
	Through breathing	2 (0.7)
	I do not know	47 (15.6)
What is the causative agent of onchocerciasis?	Black flies	216 (71.5)
	*Onchocerca volvulus*	71 (23.5)
	Plasmodium species	13 (4.3)
	I do not know	2 (0.7)
What type of treatment is used to treat onchocerciasis?	Modern medicine	279 (92.4)
	Traditional medicine	20 (6.6)
	I do not know	3 (1)
If modern, which drug is needed to treat the disease?	Ivermectin	231 (76.5)
	Metronidazole	19 (6.3)
	Other (“onch”)	52 (17.2)
Have you heard about the elimination program of onchocerciasis?	Yes	230 (76.4)
	No	71 (23.6)
From where do you get the source of information about the elimination program of onchocerciasis?	Health extension worker	192 (79.3)
	Mass media	11 (4.5)
	CDD	38 (15.7)
	Zonal health department announcement	1 (0.4)
When the information about an elimination program of onchocerciasis obtained?	Before 10 years	152 (62.6)
	Before 5 years	53 (21.8)
	After 5 years	33 (13.6)
	Not sure	5 (2.1)

**Table 4:** Attitude of community respondents about onchocerciasis from Gesha town, Ethiopia.

Indicative questions on attitude	Response category	Frequency (%)
How do you/your family perceive CDT with ivermectin?	Very useful	207 (68.5)
	Partially useful	94 (31.1)
	Not useful	1 (0.3)
Do you think the program on controlling onchocerciasis is effective?	Strongly disagree	23 (7.6)
	Disagree	15 (5.0)
	Undecided	22 (7.3)
	Agree	58 (19.2)
	Strongly agree	184 (60.9)
What do you recommend to continue the program?	Drug supply	171 (56.6)
	Incentive for CDD	76 (25.2)
	Creating community awareness	31 (10.3)
	No comment	24 (7.3)
Did the drug have any side effects?	Yes	165 (54.6)
	No	137 (45.4)

**Table 5:** Practice of the community to the success of elimination program of onchocerciasis from Gesha town, Ethiopia.

Indicative questions on practice	Response categories	Frequency (%)
The problem to follow onchocerciasis treatment properly	Yes	171 (56.6)
	No	131 (43.4)
Have all eligible family members received ivermectin properly?	Yes	198 (64.6)
	No	103 (34.6)
What is the reason to interrupt the treatment?	Fear of side effect of the drug	71 (34.6)
	Low beliefs for freely given medications	43 (21.0)
	Sign of anger of their god	26 (12.7)
	No incentive for CDD	65 (31.7)
When did you receive your last treatment?	Last year	50 (18.7)
	Before two years	36 (13.5)
	This year	181 (67.8)
Who missed the treatment?	Wife	35 (24.1)
	Husband	42 (29.0)
	Children	68 (46.9)
How many times did he/she or you miss the treatment?	One year	82 (56.9)
	Two years	42 (29.2)
	Not sure	20 (13.9)
Why did he/she or you miss treatment?	Pregnancy	50 (33.1)
	Health problem	32 (21.2)
	Not being present during drug campaign	69 (45.7)

**Table 6:** Results of the bivariate binary logistic regression analysis of knowledge, attitude, and practice towards the elimination of onchocerciasis from Gesha town, Ethiopia.

Variables	Category	COR (95% CI)	*P* Value	COR (95% CI)	*P* Value	COR (95% CI)	*P* Value
		Knowledge towards disease		Attitude towards disease		Practice towards disease	
Sex	Male	0.017 (0.008–0.035)	0.000	0.059 (0.033–0.105)	0.000	0.088 (0.051–0.152)	0.000
Age	18–25	—	—	—	—	—	—
	26–35	0.003 (0.000–0.027)	0.000	0.039 (0.015–0.101)	0.000	0.077 (0.033–0.177)	0.000
	36–45	0.008 (0.001–0.063)	0.000	0.105 (0.040–0.276)	0.465	0.085 (0.033–0.216)	0.000
	46–55	0.063 (0.007–0.548)	0.012	0.633 (0.186–2.156)	0.465	0.556 (0.188–1.640)	0.287
	56–65	0.293 (0.031–2.724)	0.280	0.667 (0.236–1.880)	0.443	0.771 (0.034–1.953)	0.583
Educational status	Degree and above	—	—	—	—	—	—
	Illiterate	0.004 (0.001–0.014)	0.000	0.045 (0.021–0.095)	0.000	0.047 (0.022–0.100)	0.000
	Primary school	0.011 (0.008–0.037)	0.000	0.050 (0.020–0.125)	0.000	0.063 (0.025–0.159)	0.000
	Secondary school	0.278 (0.001–2.976)	0.290	1.129 (0.122–10.437)	0.915	0.667 (0.113–3.934)	0.654
	Diploma	0.489 (0.125–1.919)	0.305	0.881 (0.256–2.177)	0.783	0.152 (0.493–2.690)	0.783
Occupation	Civil servant	—	—	—	—	—	—
	Farmer	0.045 (0.018–0.114)	0.000	0.162 (0.078–0.336)	0.000	0.246 (0.118–0.513)	0.000
	Merchant	0.574 (0.279–1.182)	0.132	0.758 (0.371–1.547)	0.446	1.020 (0.499–2.083)	0.957
	Student	12.83 (1.611–10.221)	0.016	2.576 (0.837–7.929)	0.099	4.185 (1.361–12.869)	0.013

**Table 7:** Multivariable binary logistic regression analysis of knowledgeable factors for the elimination of onchocerciasis from Gesha town, Ethiopia.

Variable	Category	*B*	S.E.	Wald	Sig.	Exp (*B*)	95% C.I. for EXP (*B*)	
							Lower	Upper
Sex	Male	−3.223	0.763	17.843	0.000	0.040	0.009	0.178
Age	26–35	−4.152	1.457	8.124	0.004	0.016	0.001	0.273
	36–45	−2.528	1.503	2.828	0.093	0.080	0.004	1.520
	46–55	−4.409	1.735	6.459	0.011	0.012	0.000	0.365
	56–65	−1.444	1.921	0.565	0.452	0.236	0.005	10.180
Educational status	Illiterate	−4.932	1.056	21.798	0.000	0.007	0.001	0.057
	Primary school	−2.828	0.995	8.068	0.004	0.059	0.008	0.416
	Secondary school	0.024	3.548	0.000	0.995	1.024	0.001	1071.9
	Diploma	0.082	1.196	0.005	0.945	1.086	0.104	11.327
Occupation	Civil servant	—	—	15.744	0.003	—	—	—
	Farmer	−3.471	1.067	10.577	0.001	0.031	0.004	0.252
	Merchant	−0.113	0.898	0.016	0.900	0.893	0.154	5.187
	Student	1.518	1.703	0.795	0.373	4.565	0.162	128.6
	Others	0.697	1.082	0.415	0.520	2.008	0.241	16.742
Constant	7.747	1.852	17.499	0.000	2314.	—	—	—

**Table 8:** Multivariable binary logistic regression analysis of attitude able factors for the elimination of onchocerciasis from Gesha town, Ethiopia.

Variable	Category	B	S.E	Wald	Sig.	Exp (B)	95% C.I. for EXP (B)	
							Lower	Upper
Sex	Male	−1.353	0.395	11.72	0.001	0.258	0.119	0.561
Age	18–25	—	—	10.26	0.036	—	—	—
	26–35	−1.289	0.605	4.538	0.033	0.276	0.084	0.902
	36–45	−0.208	0.641	0.105	0.745	0.812	0.231	2.855
	46–55	0.173	0.746	0.054	0.816	1.189	0.276	5.128
	56–65	−0.492	0.598	0.678	0.410	0.611	0.190	1.973
Educational status	Illiterate	−1.875	0.492	14.54	0.000	0.153	0.058	0.402
	Primary school	−1.796	0.591	9.223	0.002	0.166	0.052	0.529
	Secondary school	0.730	1.29	0.317	0.574	2.076	0.163	26.42
	Diploma	0.032	0.509	0.004	0.949	1.033	0.381	2.800
Occupation	Farmer	−0.525	0.492	1.140	0.286	0.591	0.225	1.551
	Merchant	0.183	0.512	0.128	0.721	1.201	0.440	3.278
	Student	−0.104	0.721	0.021	0.886	0.902	0.219	3.704
	Others	−0.298	0.537	0.308	0.579	0.742	0.259	2.127
Constant	2.249	0.597	14.17	0.000	9.481	—	—	

**Table 9:** Multivariable binary logistic regression analysis of practicable factors for the elimination of onchocerciasis from Gesha town, Ethiopia.

Variable	Category	*B*	S.E	Wald	Sig.	Exp (*B*)	95% C.I. for EXP (*B*)	
							Lower	Upper
Sex	Male	−0.911	0.411	4.919	0.027	0.402	0.180	0.900
Age	26–35	−0.608	0.567	1.150	0.284	0.544	0.179	1.654
	36–45	−0.674	0.602	1.257	0.262	0.509	0.157	1.657
	46–55	−0.193	0.655	0.087	0.768	0.825	0.229	2.975
	56–65	−0.390	0.526	0.549	0.459	0.677	0.242	1.898
Educational status	Illiterate	−0.074	0.479	20.59	0.000	0.114	0.045	0.292
	Primary school	−1.918	0.562	0.023	0.16	1.076	0.407	2.844
	Secondary school	−0.119	0.953	0.016	0.901	0.888	0.137	5.753
	Diploma	−1.918	0.562	11.65	0.001	0.147	0.049	0.442
Occupation	Civil servant	0.215	0.456	0.223	0.637	1.240	0.507	3.034
	Farmer	0.074	0.496	0.022	0.882	1.076	0.407	2.844
	Merchant	0.562	0.485	1.342	0.247	1.754	0.678	4.538
	Student	0.686	0.681	1.015	0.314	1.987	0.523	7.552
	Others	0.260	0.484	0.289	0.591	1.297	0.502	3.351
Constant	1.275	0.503	6.423	0.011	3.577			

## Data Availability

All data generated or analyzed throughout the study are fully available in this article. However, raw data can be obtained from the corresponding author on reasonable request.
